# The Influence of Chronic Wound Extracts on Inflammatory Cytokine and Histatin Stability

**DOI:** 10.1371/journal.pone.0152613

**Published:** 2016-03-28

**Authors:** Mireille A. Boink, Sanne Roffel, Kamran Nazmi, Catherine van Montfrans, Jan G. M. Bolscher, Amit Gefen, Enno C. I. Veerman, Susan Gibbs

**Affiliations:** 1 Department of Oral Biochemistry, Academic Centre for Dentistry Amsterdam (ACTA), University of Amsterdam and VU University Amsterdam, Amsterdam, The Netherlands; 2 Department of Dermatology, VU University medical center, Amsterdam, The Netherlands; 3 Department of Biomedical Engineering, Faculty of Engineering, Tel Aviv University, Tel Aviv, Israel; 4 Department of Oral Cell Biology, Academic Centre for Dentistry Amsterdam (ACTA), University of Amsterdam and VU University, MOVE Research Institute Amsterdam, Amsterdam, The Netherlands; University of North Texas Health Science Center, UNITED STATES

## Abstract

Chronic ulcers represent a major health burden in our society. Despite many available therapies, a large number of ulcers do not heal. Protein based therapies fail in part due to proteolytic activity in the chronic wound bed. The aim of this *in vitro* study was to determine whether typical inflammatory cytokines and human salivary histatins remain stable when incubated with chronic wound extracts. Furthermore we determined whether a short exposure of histatins or cytokines was sufficient to exert long term effects on fibroblast migration. Stability of human recombinant cytokines IL-6 and CXCL8, and histatin variants (Hst1, Hst2, cyclic Hst1, minimal active domain of Hst1) in the presence of chronic wound extracts isolated from non-healing ulcers, was monitored by capillary zone electrophoresis. Migration-stimulating activity was assessed using a dermal fibroblast wound healing scratch assay. Histatins and cytokines stayed stable in saline for > 24h at 37°C, making them ideal as an off-the-shelf product. However, incubation with chronic wound extracts resulted in serious breakdown of Hst1 and Hst2 (~50% in 8h) and to lesser extent cyclic Hst1 and the minimal active domain of Hst1 (~20% in 8h). The cytokines IL-6 and CXCL8 were more stable in chronic wound extracts (~40% degradation in 96h). An initial 8-hour pulse of histatins or cytokines during a 96-hour study period was sufficient to stimulate fibroblast migration equally well as a continuous 96-hour exposure, indicating that they may possibly be used as novel bioactive therapeutics, exerting their activity for up to four days after a single exposure.

## Introduction

Chronic ulcers represent a major health burden in our society and significantly impair the quality of life of millions of people [1]. Chronic ulcers are wounds that have no tendency to heal within three months, despite optimal treatment [2;3]. The prevalence of chronic wounds is approximately 1% of the population. The incidence is expected to increase dramatically in the near future due to ageing of the population and to an increased prevalence of health-related disorders, such as vascular diseases (venous and arterial ulcers) and diabetes (diabetic foot ulcers) [2]. Even though 50% of treated venous leg ulcers will heal within four months, the prognosis is not promising since recurrence rates are as high as 26%-56%. The majority of recurrences occurs within the first three months after initial healing [4]. Therefore, optimal treatment of ulcers is of a high socioeconomic significance. For example, in a prospective cost-of-illness study the total medical costs of non-healing ulcers in Germany per patient and year amount to more than € 9000 [5].

Therefore, novel strategies for chronic wound treatment are required. Ideally, these should be easily applicable and combinable with current standard therapies such as compression therapy for venous ulcers and pressure offloading for diabetic foot ulcers. Moreover, the new therapeutic should be stable and should not be degraded too fast by other factors present in the chronic wound bed. Over the years a number of growth factors were studied for their healing capacities, such as recombinant human (rh) TGFβ, GM-CSF, PDGF, KGF and autologous platelet lysate. However, none of these individual factors have yet been proven to be effective for chronic ulcer treatment [6].

Many cytokines have been identified as key players in directly stimulating wound closure by enhancing proliferation and migration of keratinocytes, fibroblasts and endothelial cells [7;8]. For example, CXCL8 stimulates human keratinocyte migration and proliferation as well as angiogenesis [9–11]. In mice, a key role for IL-6 was implicated by the finding that wound healing was severely delayed in IL-6 knockout mice as shown by lower leukocyte infiltration, granulation tissue formation, re-epithelialisation and angiogenesis [12–14]. Therefore, these cytokines are promising candidates for new topical therapeutics.

Other candidates for a novel topical ulcer therapeutic are histatins. We have shown previously that histatins (Hst), a family of peptides which are specifically secreted in saliva of higher primates, are the predominant factors in human saliva responsible for oral keratinocyte and fibroblast migration [15]. This strongly implicates their role in oral wound healing. Hst3 and 5 are known for a long time to possess antimicrobial activities [16;17] whereas Hst1 and 2 have been found to enhance wound closure *in vitro* [15;18;19]. We have identified the minimal active domain of Hst1 –SHREFPFYGDYGS (mad-Hst1)–that is responsible for stimulating keratinocyte migration [20]. Since cyclic peptides are generally more stable than linear peptides, a cyclic variant of Hst1 (cHst1) was synthesized. Thus, besides Hst1 and Hst2, which are the naturally in saliva occurring peptides with migration-stimulating activity, we tested cHst1 and mad-Hst1.

The chronic wound bed is known to contain many proteases which are thought to be in part responsible for the inability of ulcers to heal. Such proteases have been shown to break down extracellular matrix proteins such as collagen [21]. Therefore when a protein based therapeutic (e.g. peptide or cytokine) is applied to a wound bed to stimulate healing, it will have to exert its function despite the presence of many proteases that possibly degrade it over time. In this study we investigated the stability of cytokines (IL-6 and CXCL8) and four synthetic histatins in a chronic wound environment. This environment was simulated by incubation of the cytokines and peptides *in vitro* with chronic wound extracts (CWE) obtained from wound debridement material of patients with therapy-resistant leg ulcers. The migration-stimulating activity *in vitro* was determined in a wound healing scratch assay using dermal fibroblasts.

In this study we investigated whether the *ex vivo* proteolytic breakdown (inactivation) in CWE of the six migration-stimulating peptides would be severe enough to significantly interfere with peptide activity. If these cytokines and histatins survive, at least to some extent, the hostile proteolytic environment in chronic ulcers, further investigation as topical therapeutics is indicated.

## Materials and Methods

### Human tissue

Human tissue (skin and chronic wound debridement material) was obtained after oral informed consent and used in an anonymous fashion in accordance with the ‘Code for Proper Use of Human Tissues’ as formulated by the Dutch Federation of Medical Scientific Organizations (www.fmwv.nl) and following procedures approved by the institutional review board of the VU University medical center. According to the Dutch law, neither approval of an ethics committee nor written consent of the patients is required when using surgical waste material.

### Isolation and culture of dermal fibroblasts

Dermal fibroblasts were isolated from healthy human abdominal skin from patients undergoing corrective abdominal plastic surgery. After removal of all adipose tissue, the skin was washed in PBS and incubated overnight on dispase II (Roche, Mannheim, Germany) at 4°C. The next day, epidermis was removed and discarded and the dermis was incubated in collagenase type II (Gibco, Life Technologies, Grand Island, USA)/dispase II in Hanks balanced salt solution (HBSS) (Gibco) at 37°C for 2–3 h. Fibroblast medium consisting of DMEM (Lonza, Verviers, Belgium) containing 1% ultroserG (UG) (Biosepra, Cergy-Saint-Christopher, France) and 1% penicillin-streptomycin (P/S) (Gibco) was added and the solution was passed through an 100 μm and 40 μm cell strainer (Becton Dickinson Falcon, Erembodegem, Belgium). A single cell suspension was seeded at ± 3.5 x 10^4^ cells cm^2^ and further cultured in fibroblast medium. Medium was changed twice a week and cultures were passaged when 90% confluent using 0.5mM EDTA/0.05% trypsin (Gibco). The cells were maintained at 37°C in a humidified atmosphere containing 5% CO_2_ and used for experiments at passage 3.

### Preparation of chronic wound extracts

Debridement tissue was obtained from chronic ulcers of 15 patients with therapy resistant (arterio-venous) leg ulcers, according to procedures described earlier [22]. In brief, moist, easily removable debridement material, essentially blood free, was collected from the ulcer surface and divided equally into two different solutions: (i) PBS (B. Braun, Melsungen, Germany), (ii) PBS with protease inhibitor cocktail (PIC) (1:100; v/v) (Sigma-Aldrich, Steinheim, Germany). The mixtures were gently shaken for 1 h at 4°C to elute proteins and centrifuged; the supernatants were stored at -80°C. Total protein content of the supernatant was measured using the BioRad Protein Assay (BioRad Laboratories, Hercules, California, USA) essentially as described by the supplier. Samples were diluted to 1 mg/ml protein and extracts of five different donors were pooled; three different pools of five donors were used in all experiments. The samples are referred to as chronic wound extracts (CWE).

### Peptides and cytokines

Linear histatin peptides (Table 1) were manufactured by solid phase peptide synthesis using 9-fluorenylmethoxycarbonyl (Fmoc)-chemistry with a MilliGen 9050 synthesizer (Milligen-Biosearch, Bedford, MA, USA) according to the manufacturer’s protocols. Peptide synthesis grade solvents were obtained from Actu-All Chemicals (Oss, The Netherlands), the preloaded Nova SynTGA resins from Nova Biochem (Merck Schuchardt, Hohenbrunn, Germany) and the N-α-Fmoc-aminoacids from Orpegen-Pharma (Heidelberg, Germany) and Iris Biotech (Marktredwitz, Germany). Peptides were purified by preparative RP-HPLC on a Dionex Ultimate 3000 system (Thermo Scientific, Breda, The Netherlands) with a Grace Spring column of 250 mm x 25 mm (Grace, Deerfield, IL, USA) containing Vydac C18 TP beads 10 μm (Hesperia, CA,USA). Elution was performed with a linear gradient from 20 to 45% acetonitrile (AcN) containing 0.1% trifluoroacetic acid (TFA) in 20 min at a flow rate of 20 ml/min. The absorbance of the column effluent was monitored at 214 nm, and peak fractions were pooled and lyophilized. Reanalysis by RP-HPLC on an analytic Vydac C18-column (218MS54) developed with a similar gradient at a flow rate of 1 ml/min revealed a purity of at least 95%. The authenticity was confirmed by mass spectrometry with a Microflex LRF MALDI-TOF, equipped with a gridless reflectron (Bruker Daltonik GmbH, Bremen, Germany) as previously described [23].

**Table 1:** Peptide sequences.

Peptide	Amino acid sequence	MW (Da)
Hst1	DSHEKRHHGYRRKFHEKHHSHREFPFYGDYGSNYLYDN	4848
c-Hst1	GGDSHEKRHHGYRRKFHEKHHSHREFPFYGDYGSNYLYDNLPET	5383
Hst2	RKFHEKHHSHREFPFYGDYGSNYLYDN	3445
Mad-Hst1	SHREFPFYGDYGS	1560

Sortase-catalyzed cyclization of Hst1 (Table 1) was performed as described previously [23]. Briefly, linear Hst1 equipped with the sortase A cleavage site LPETGG at the C-terminus and a diglycine motif (GG) at the N-terminus allowing intra-molecular ligation, was synthesized as described above. The head-to-tail ligation was conducted with 0.5 mM of the linear peptide and 50μM sortase A in 50 mM Trisbuffer, pH 7.5, containing 150 mM NaCl and 10 mM CaCl_2_ at 37°C for 24 h. The cHst1 was purified from the reaction mixture by preparative RP-HPLC as described above.

RhIL-6 was purchased both from Biovision Inc, Milpitas, CA, USA (Cat nr. 4143) and Cell Systems Biotechnology, Troisdorf, Germany (Cat nr. CS-C1066) and rhCXCL8 from both R&D Systems, Abingdon, United Kingdom (Cat nr. 208-IL) and Cell Systems (Cat nr. CS-C1069).

### Determination of stability in CWE using capillary zone electrophoresis and HPLC

To determine the stability of the different peptides in CWE, histatins and cytokines were dissolved in PBS at 1 mg/ml. Histatins and cytokines were incubated in a 1:1 ratio with (i) PBS, (ii) CWE in PBS, or (iii) CWE in PBS containing PIC, in a total volume of 25 μl. Imidazole (Sigma-Aldrich) was added as an internal standard, with a final concentration of 100 μg/ml. The mixtures were incubated at 37°C, and samples were taken at 0, 24, 48, 72 and 96 h. For histatins containing mixtures, additional samples were taken after 2, 4, 8 and 16 h of incubation.

For the dose response of PIC and Hst1, three different concentrations of Hst1 were prepared (0.5 mg/ml, 1 mg/ml and 2 mg/ml) and two different concentrations of PIC were used. The final PIC concentration in the CWE (1 mg/ml) sample was 1:140 v/v (same as above) or 1:280 v/v (2x diluted). Mixtures were incubated at 37°C and samples were taken at 0, 24 and 96 h.

The samples were analyzed with a BioFocus 2000 capillary zone electrophoresis system (Bio-Rad Laboratories, Hercules, CA, USA) equipped with an uncoated fused-silica capillary of internal diameter 50 μm and a length of 24 cm. Samples were loaded by pressure injection (5 psi/sec). Separation was performed at 10 kV (cathode at the detector side) at 20°C using 0.1 M phosphate buffer (pH 2.5) as electrolyte and monitored continuously at 200 nm. Peaks were quantified with the BioFocus Integrator software. In order to quantify the percentage of intact peptides remaining after incubation with CWE, the ratios of histatins, rhIL-6 or rhCXCL8 to internal standard was set at 100% at 0h, and the ratios at the other incubation time points were related to this.

The histatins containing mixtures were also analyzed by RP-HPLC on an analytic Vydac C18-column (218MS54) (Grace, Columbia, Maryland, USA). Elution was performed with a linear gradient from 20 to 40% AcN containing 0.1% TFA in 25 min at a flow rate of 1 ml/min. The absorbance of the column effluent was monitored at 214 nm. Peak fractions were collected and analyzed by mass spectrometry with a Microflex LRF MALDI-TOF.

### Assessment of migration-stimulating activity, using fibroblast wound healing scratch assays

A wound healing scratch assay was performed to determine the effect of histatins, rhIL-6 and rhCXCL8 on fibroblast migration. Confluent monolayers of fibroblasts in a 48-well plate (Costar, Corning Incorporated, New York, USA) were incubated with serum-free medium (DMEM containing 1% P/S and 0.1% bovine serum albumin (Sigma-Aldrich)) for 96 h to ensure minimum spontaneous migration. Next, a scratch was drawn with a disposable plastic pipette tip. Cultures were exposed to Hst1, cHst1, Hst2 and mad-Hst1 (all used at a concentration of 5μM), rhIL-6 (125 ng/ml), rhCXCL8 (250 ng/ml) and vehicle (H_2_O) in serum free medium. Two experimental set-ups were studied: i) cells were exposed only during the first 8 h to histatins and cytokines and then transferred to vehicle supplemented medium for an additional 88 h; or ii) cells were exposed to histatins and cytokines during the entire 96 h study period. Phase contrast micrographs were taken directly after drawing the scratch and after four days (96 h) of exposure. Data were analyzed using an image processing algorithm that has been described in detail in our previous work [24–26]. The area of the scratch covered by migrating fibroblasts was determined using the following equation: (E_0_-E_96_)/(U_0_-U_96_), in which: E = exposure to a condition (IL-6, CXCL8 and histatins); U = unexposed (serum-free medium); E_0_ and U_0_ = area of the scratch at T = 0 h; E_96_ and U_96_ = area of the scratch (not covered with cells) after 96 h. In this way, in each exposed group (IL-6, CXCL8, histatins) the resurfaced area at 96 h is compared to its own 0 h time point and then set relative to the unexposed group (serum-free medium).

### Statistics

All data are presented as mean ± standard error mean. Differences in breakdown of histatins and cytokines in CWE after different times of incubation were compared with T = 0 by Friedman one-way ANOVA with Dunn’s multiple comparisons test. Differences between presence and absence, or differences between dilutions of protease inhibitor incubation in the stability test of histatins and cytokines in CWE were analyzed by two-way ANOVA with Sidak’s multiple comparisons test. In the migration experiments differences between 8 h and 96 h exposure and between exposed and unexposed were also evaluated by two-way ANOVA with Sidak’s multiple comparisons test. Statistics were calculated in GraphPad Prism (San Diego, CA, USA). Differences were considered significant when *P < 0.05, **P < 0.01, ***P <0.005.

## Results

### Stability of histatins, rhIL-6 and rhCXCL8 in chronic wound extracts

**Fig 1:**
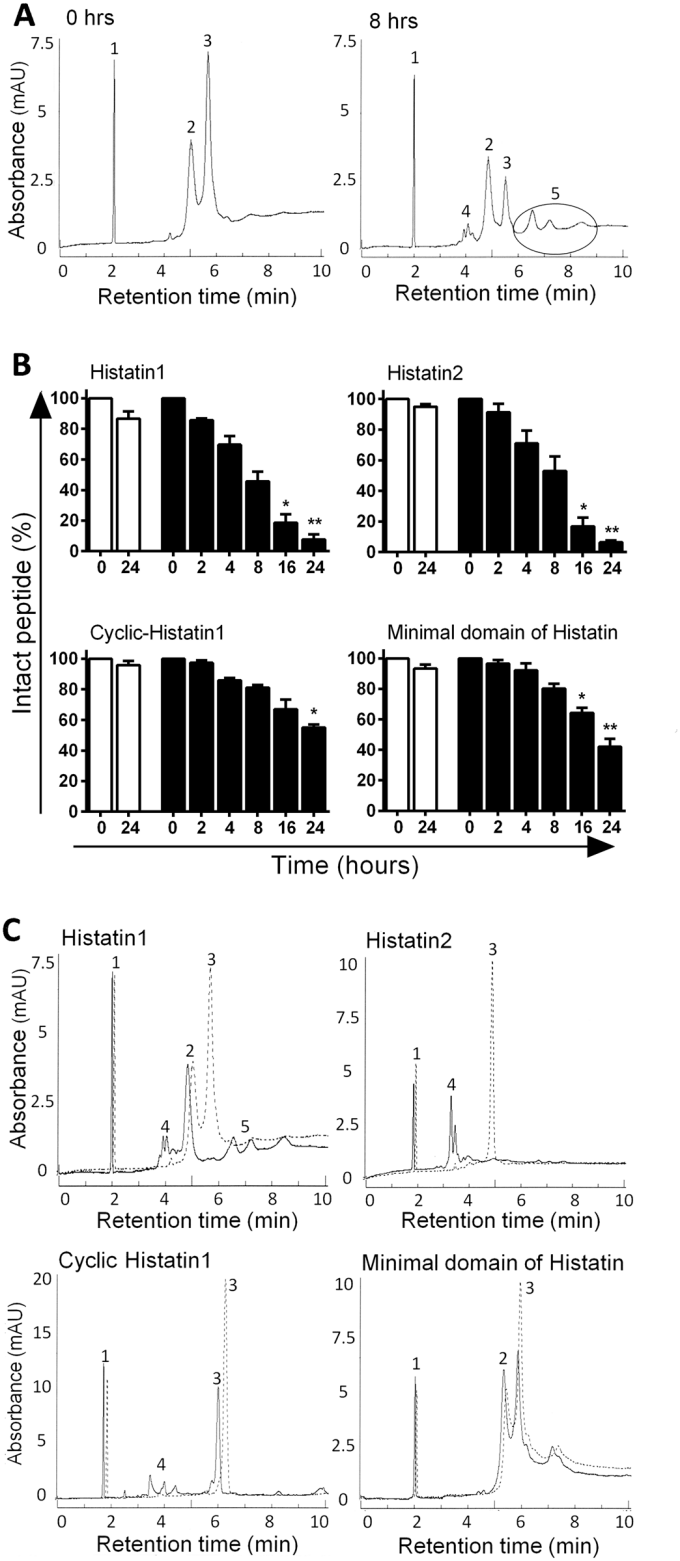
Stability of histatins during incubation with chronic wound extract. A: Capillary zone electrophoresis graph of Hst1 incubation with chronic wound extracts at 0 and 8 h. At 0 h three peaks are visible: 1 = internal standard, 2 = CWE, 3 = Intact Hst1. At 8 h two additional peaks arise, representing breakdown products of Hst1 and CWE (peak 4 and 5). B: Percentage of intact histatins after incubation with CWE, relative to the amount at the start of incubation. White bars = incubation with PBS, black bars = incubation with CWE. Bars represent means ± SEM of three independent experiments, performed with CWE pools of 5 donors. * P <0.05, ** P <0.01. C: Capillary zone electrophoresis graph of histatin incubation with CWE; dashed line = 0 h, solid line = 24 h. For a description of the peak number see legend A.

Protein-based therapeutics for stimulation of wound healing (e.g. histatins or cytokine) are intrinsically sensitive to the multitude of proteases that are present in the chronic wound bed. To get an impression whether Hst1, cHst1, Hst2 and mad-Hst1 as well as rhIL-6 and rhCXCL8 would survive long enough in chronic ulcers to exert their migration-stimulating activity, we tested their stability in diluted CWE *in vitro*. Proteolytic breakdown was monitored by capillary zone electrophoresis. Hst1 incubation with CWE revealed that at 0 h three peaks are visible (Fig 1A), representing the internal standard (imidazole), CWE and Hst1. After 8 h of incubation in CWE with imidazole the Hst1 peak decreased and additional peaks indicate the presence of degradation products of Hst1.

All histatins proved stable in PBS for at least 24 h (Fig 1B). In the presence of CWE Hst1 and Hst2 were readily degraded until 46% and 53% respectively of the original amount of peptide remained intact after 8 h and only 8% and 6% after 24 h (Fig 1B and 1C). Notably, cHst1 and mad-Hst1 were much more stable, with 81% of cHst1 and 80% of mad-Hst1 still being intact after 8 h and 55% and 42% after 24 h.

**Fig 2:**
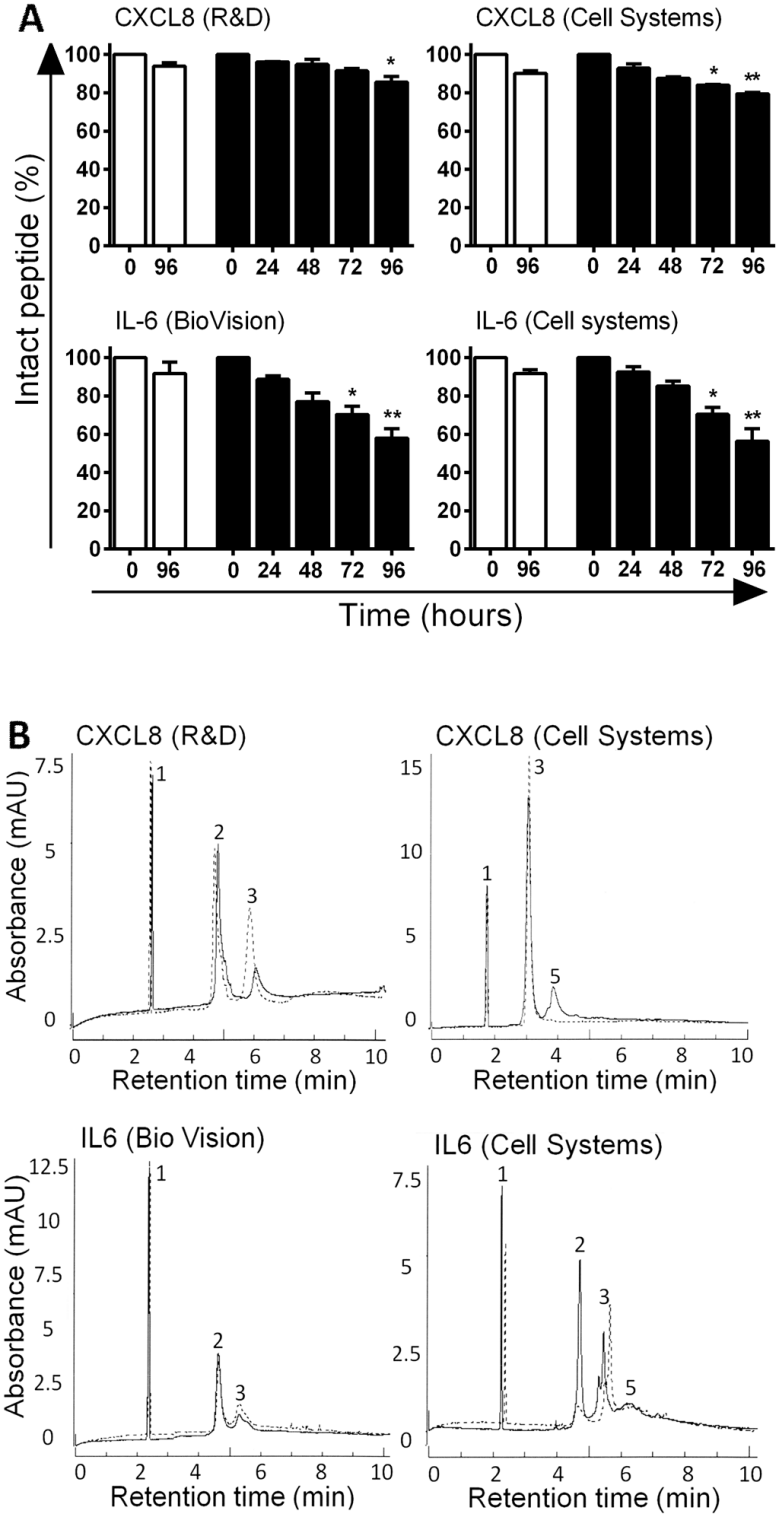
Stability of cytokines during incubation with chronic wound extract. A: Percentage of intact cytokines during incubation with CWE, relative to the cytokine amount at the start of incubation. White bars = incubation with PBS, black bars = incubation with CWE. Bars represent means ± SEM of three independent experiments, performed with CWE pools of 5 donors. * P <0.05, ** P <0.01. B: Capillary zone electrophoresis graph of cytokine incubation with CWE; dashed line = 0 h, solid line = 96 h. 1 = internal standard, 2 = CWE, 3 = Intact cytokine, 4 + 5 = degradation products.

Compared to the histatins, the cytokines tested were relatively stable in the presence of CWE. Even after an extended incubation period of 96 h with CWE, for rhCXCL8 approximately 80% of the starting material remained intact, with small differences with respect to the source (R&D: 86%, Cell Systems: 79%) and for rhIL-6 more than 55% remained intact (Biovision 58%; Cell Systems 56%) (Fig 2).

**Fig 3:**
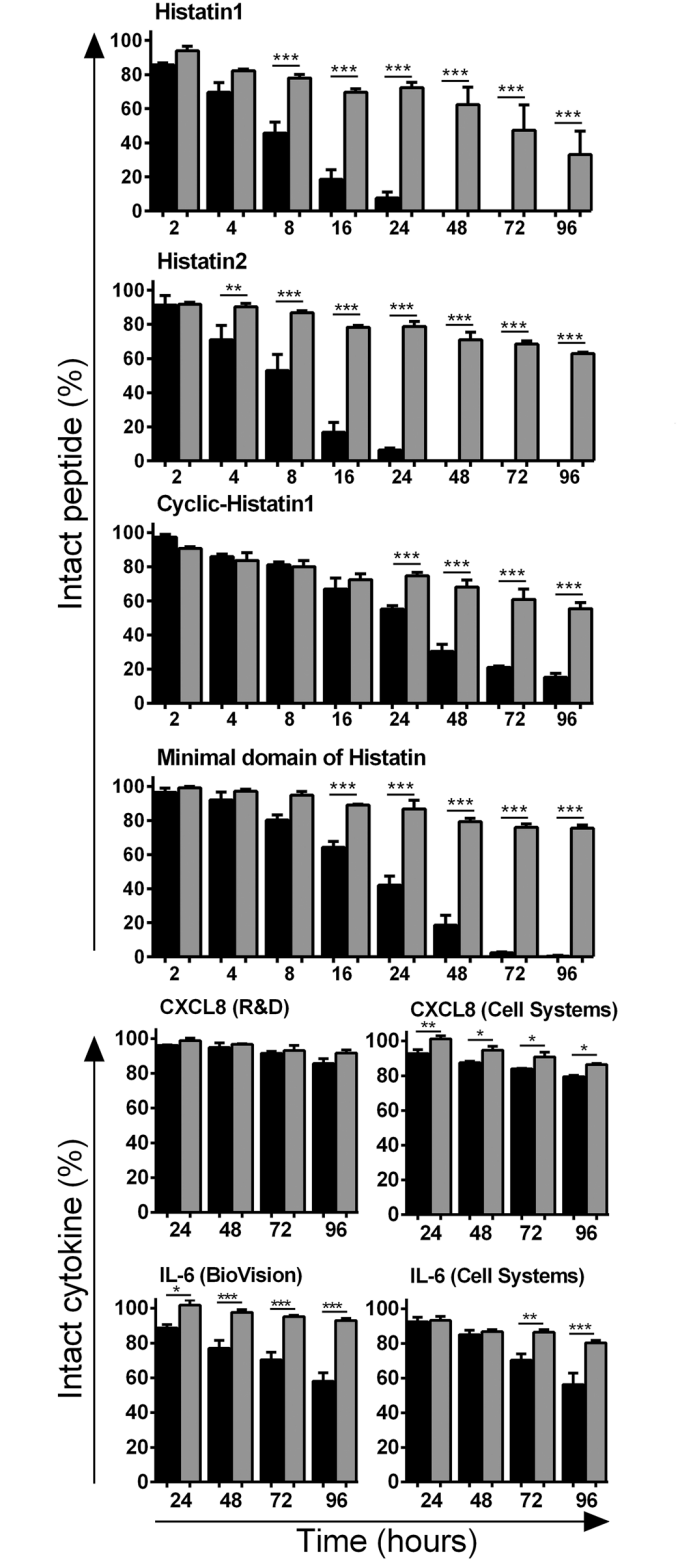
Protease inhibitors prevent breakdown of histatins and cytokines during incubation with chronic wound extract. Degradation of histatins and cytokines during incubation with CWE, with or without addition of protease inhibitors, as percentage of the start amount. Bars represent means ± SEM of three independent experiments, performed with CWE pools of 5 donors. Black bars = incubation with CWE, grey bars = incubation with CWE + PIC. * P <0.05, ** P <0.01, *** P <0.005.

Degradation of histatins diminished significantly by addition of a protease inhibitor cocktail (PIC) to CWE (Fig 3). For Hst1 and Hst2, the remaining fraction of intact peptide after 24 h rose to 72% and 78% respectively. Similar results were obtained for cHst1 and mad-Hst1 which rose to 74% and 87% respectively. For cytokines, the degradation was diminished in the presence of PIC, with remaining fractions being 80% to 93% (Fig 3).

**Fig 4:**
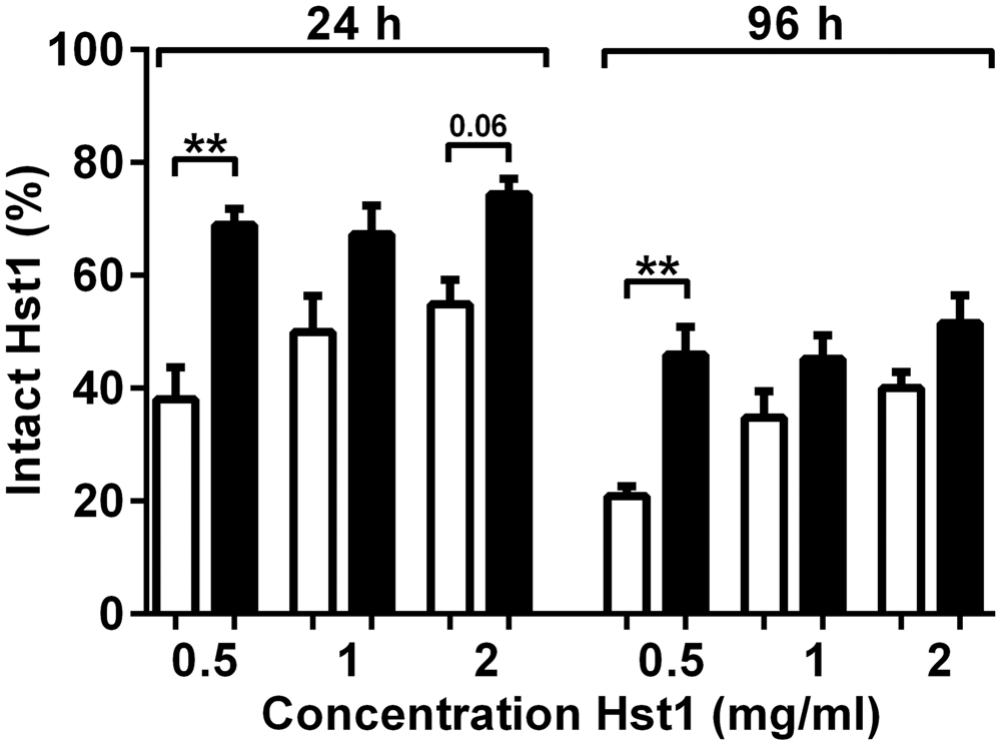
Dose response of Hst1 and PIC in the presence of CWE. Percentage of intact Hst1 remaining after incubation with different dilutions of PIC (1:140 and 1:280) in CWE (1 mg/ml). Starting concentrations of Hst1 were 0.5, 1 and 2 mg/ml. Bars represent means ± SEM of three independent experiments, performed with CWE pools of 5 donors. Black bars = PIC:CWE 1:140 v/v, white bars = PIC:CWE 1:280 v/v. * P <0.05, ** P <0.01.

Since PIC clearly prevented breakdown of the peptides, a dose response was next performed to identify the minimum amount of Hst1 peptide and PIC inhibitor necessary to still prevent peptide breakdown in the presence of CWE. Such an experiment is of relevance when considering a future clinical minimum dose. Hst1 was used at 0.5, 1 and 2 mg/ml. The final PIC concentration in the CWE sample was 1:140 v/v (same as in Fig 3) or 1:280 v/v (2x diluted). Degradation of Hst1 was assessed after 24 and 96 h. The higher concentration of PIC (1:140) resulted in increased stability of Hst1 at all three concentration compared to the 1:280 diluted PIC (Fig 4). The lower concentration of Hst1 (0.5 mg/ml) showed a trend to increased degradation at both PIC concentrations. Therefore it can be concluded that the concentrations of Hst1 (1 mg/ml) and PIC (1:140 v/v) used in Fig 3 were optimal for the *in vitro* experiments.

### Migration-stimulating activity of histatins, rhIL-6 and rhCXCL8

**Fig 5:**
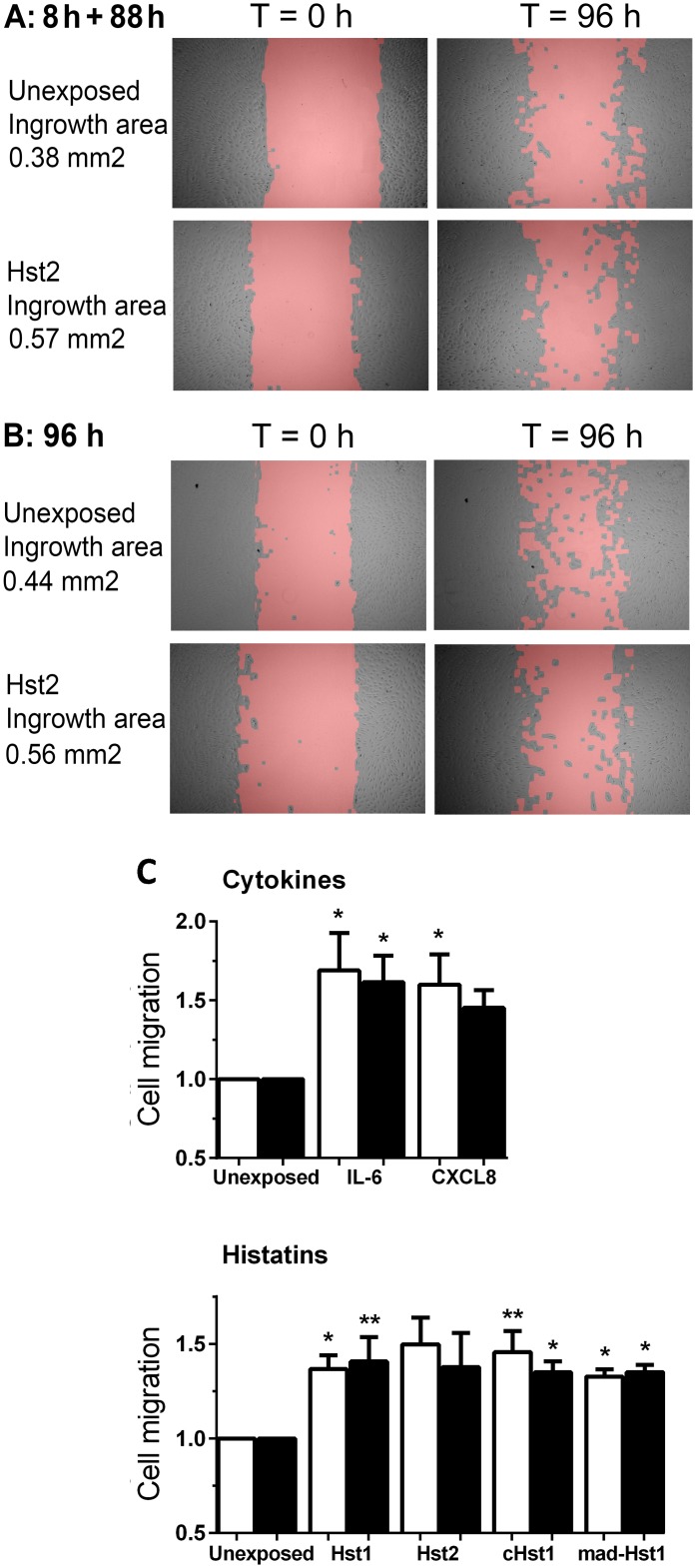
An 8 h pulse of peptides stimulates fibroblast migration to the same extent as continuous 96 h exposure. Migration of dermal fibroblasts in a scratch wound healing assay is shown. At time of introduction of the scratch into a confluent layer of dermal fibroblasts, cultures were supplemented with the peptides Hst1 (5 μM), Hst2 (5 μM), cHst1 (5 μM), mad-Hst1 (5 μM), rhIL-6 (125 ng/ml), rhCXCL8 (250 ng/ml) and vehicle (H_2_O). A, B: Representative photographs of fibroblasts in the scratch wound healing assay at 0 h (left pictures) and 96 h (right pictures) after culture. Cells were unexposed (upper two pictures) or exposed to Hst2 (lower two pictures) for 96 h (A) or for 8 h after which medium was changed to peptide free medium for the remaining 88 h (B). C: Migration in the scratch wound healing assay. White bars = 8 h exposure with peptides, followed by 88 h incubation without peptides, black bars = 96 h continuous exposure with peptides. Bars represent means ± SEM of three independent experiments each performed in quadruplicate. * P <0.05, ** P <0.01, *** P <0.005: comparison between exposure and control; no significant differences found between 8 h and 96 h of exposure. At 96 h the extent of migration (area covered) was determined with an image processing algorithm and each culture was compared to its own 0 h time point and then set relative to the unexposed group (see extensive description in Materials and Methods).

Having established that histatins are indeed susceptible to proteolytic degradation by proteases in chronic wound extracts and therefore have a limited physiological half-life, we were left with a question that is crucial for their migration-stimulating activity: ‘Is a significant concentration of histatins essential during the entire migration process, or do histatins act as signalling or initiating molecules that are only necessary to set the process in motion?’ In the latter case, prolonged exposure to histatins would not be necessary and degradation is less critical. To answer this question we tested whether a short exposure of histatins or cytokines was sufficient to exert a long term effect on fibroblast migration in the scratch assay. After an initial exposure of 8 h, before significant degradation begins to occur for cHst1, mad-Hst1, rhIL-6 and rhCXCL8, and where less than 50% of Hst1 and 2 is degraded, fibroblasts were cultured for an additional 88 h culture in the absence of either histatins or cytokines. Fibroblast migration into the scratch area was determined and compared with migration after continuous incubation (96 h) to histatins and cytokines (Fig 5). Notably, the initial 8 h pulse of either histatins or cytokines during a 96 h study period resulted in a moderate (approximately 1.4 fold) increase in cell migration into the scratch area compared to unexposed fibroblasts and this was not significantly different to that observed after a continuous 96 h exposure. This indicates that these potential wound healing mediators exerted their maximum effect on fibroblast migration within 8 h after administration, which was then sustained after their removal for up to 96 h, demonstrating that prolonged exposure to either intact histatins or cytokines is not essential.

## Discussion

Our results show that histatins and cytokines are stable in PBS for at least 24 h and 96 h respectively at 37°C. This makes them ideal for easy storage as an off-the-shelf product. Notably, no extreme peptide degradation was observed within the first 8 h of incubation with CWE. Also in a scratch assay, an initial 8 h pulse of peptides during a 96 h study period resulted in the same amount of fibroblast migration as a continuous 96 h exposure to these peptides. Taken together these results indicate that histatins and cytokines may possibly be used in the future as novel bioactive therapeutics which exert their migration-stimulating activity for up to 4 days after a single application. Probably, they are best used in combination with standard therapy. Extended incubation with CWE resulted in degradation of histatins and cytokines, which could be diminished by addition of protease inhibitor cocktail (PIC). The most abundant proteases in CWE are matrix metalloproteinases (MMPs) [27]. MMPs have been described to play a role in all phases of wound healing, by cleaving and processing cytokines to increase or decrease their activity. MMPs can promote re-epithelialization and have been described to degrade and remodel the provisional extracellular matrix components as well [21]. In chronic wounds there is an imbalance between the level and activity of proteases and their inhibitors, leading to an imbalance of extracellular matrix degradation and deposition, which is associated with impaired wound healing [28]. MMPs generally reduce chemokine activity. This is especially true for CC chemokines; for CXC chemokines however, the response to MMP cleavage is more varied. CXCL1, 2 and 3 for example are completely resistant to cleavage, while both CXCL5 and CXCL8 have been described to become more active upon truncation, and CXCL12 activity is decreased upon cleavage [21]. CXCL8 (1–77) and CXCL8 (6–77) were characterized as the major forms derived from endothelial cells or fibroblasts and leukocytes respectively [29] and these were used in our study: CXCL8 (1–77) from Cell Systems and CXCL8 (6–77) from R&D where both used in stability tests, whereas CXCL8 (6–77) from R&D was used in the migration scratch assay. In contrast, no information was available for IL-6 regarding resistance to proteases.

The stability of the different histatin variants differed considerably. In this study we show that the cyclic form of Hst1 is much more stable than the linear form in CWE. Cleavage of cHst1 first needs the action of an endoprotease before the exoproteases can add in. Also, Hst1 and Hst2 have more cleavage sites than the much smaller mad-Hst1. However, it is very possible that the minimal active domain of Hst1 and Hst2 is not cleaved and that the observed degradation occurs in the flanking regions. Since cHst1 and mad-Hst1 are more stable in CWE than Hst1 and Hst2, from a the clinical point of view these would be preferred over the linear peptides as a minimum amount of drugs would be required. In our *in vitro* study it was technically not possible to perform the scratch assay with the different peptides in the presence of CWE as the CWE was limiting for such extensive studies. Furthermore, CWE contains many peptides itself which we have previously shown to be bioactive [22] and which would confound the readout of the scratch assay.

Notably, our results show that rhIL-6 and rhCXCL8 stayed relatively stable in CWE for 96 h at 37°C. There is a significant difference between histatins and the cytokines rhIL-6 and rhCXCL8 in terms of stability in CWE, which could be related to differences in their chemical structure. Histatins are linear peptides, whereas cytokines have a complex constrained chemical structure. Both IL-6 and CXCL8 contain two disulfide bonds which are very important for their tertiary structure. It is possible that these cytokines are cleaved by proteases, but this was not detected by capillary electrophoresis data used in our study, which separates based on charge, and would therefore not detect the different spliced parts which are held together by disulfide bonds. We hypothesize that cleavage within these cytokines will not reduce their activity as long as the peptide is held together by disulfide bonds, but more research is necessary to determine if this actually is the case.

With regards to cytotoxicity, we clearly show that intact cytokines and histatins are not cytotoxic during the 96 h scratch assay. It was technically not possible to determine the cytotoxicity of the individual degradation products formed in CWE. However, in the past we have tested many different synthetic histatin peptide fragments and none were found to be cytotoxic to cells in a scratch assay [20]. Also, we have tested CWE at a concentration of up to 10% in a fibroblast scratch assay and in culture medium of skin equivalents with no cytotoxicity being apparent [22]. Therefore we can conclude that the histatin breakdown products and CWE are not cytotoxic. It has also been described that persistent inflammation is observed in chronic wounds but it is unclear whether the inflammation is a cause or a result of delayed wound healing [22]. Such an inflammation may be associated with increased IL-6 and CXCL8. However, it is unknown whether the IL-6 and CXCL8, which can be detected by ELISA in CWE is biologically active or not and therefore it is currently unknown what the added benefit of applying additional cytokines e.g. IL-6 or CXCL8 to the chronic wound would be [22].

Our results give an indication of the mode of action and expected efficacy of histatins and cytokines, which can form the basis of designing a clinical phase 1 study. Recently we have shown that histatins promote cell attachment and spreading as well as cell migration [30]. In the present study we aimed to mimic explicitly, as far as possible, proteolytic degradation aspects as present in a wound, in which the peptides remain stable for a limited amount of time in the hostile wound environment. This was found to be approximately 8 hours. In the wound healing scratch assay the morphology of the cells during the 96 hour study period was unaltered indicating that the repair is stable. Furthermore we have recently reported that prolonged treatment with Hst1 (>5 days) improved cell-cell adhesion. This effect was maintained for up to 15 days thereafter [30]. Moreover, Hst2 enhanced re-epithelialization after wounding in organotypic skin equivalents when supplemented to the culture medium for 6 days [20]. Together, this suggests that histatin has a dual function in wound healing: in the initial phase it stimulates the migration of cells into the wound bed, whereas in the later phase it enhances the cell-cell adhesion leading to restoration of tissue integrity.

Care has to be taken when interpreting *in vitro* experiments and how the data can be extrapolated to the clinical situation. In contrast to our study, in clinical practice the debrided wound bed would be a non-diluted protease-rich environment and therefore in a phase 1 study further optimization on the *in vivo* peptide dose finding will be very important. Furthermore, as already mentioned, it is not expected that these peptides would form a stand-alone therapy, but would be used in combination with e.g. standard therapy, since wound healing is a complex dynamic process. In this study we used primary healthy fibroblasts, but in many difficult-to-heal ulcers there is a senescent cell population [31;32]. Another factor related to difficult-to-heal ulcers is hypoxia. Although temporary hypoxia after injury may be beneficial for cell migration, angiogenesis and growth factor production, prolonged hypoxia is not [33]. Sustained oxidative stress prolongs inflammation in chronic wounds, and impairs migration and proliferation of dermal fibroblasts and keratinocytes [34]. In a follow-up *in vitro* study, it would be important to compare fibroblasts derived from healthy skin with those derived from chronic wounds of different pathologies e.g. venous arterial ulcers and diabetic foot ulcers and also to investigate the effects of chronic hypoxia. Furthermore it will be necessary to compare CWE from the healing ulcer to that of the therapy resistant ulcer since ulcer healing is a dynamic process. For example, in normal wound healing, cytokines such as IL-6 are pleiotrophic in nature, exhibiting increases and decreases according to the homeostatic environment. The benefit of adding any peptide to the chronic wound will probably depend on timing. This needs to be taken into account when developing therapies which involve adding extraneous healing factors to the chronic wound as it is very difficult to mimic the bodies capacity to secrete or inhibit secretion of factors according to the physiological needs.

## Supporting Information

S1 Fig Stability of histatins during incubation with chronic wound extract. (XLSX)

S2 Fig Stability of cytokines during incubation with chronic wound extract. (XLSX)

S3 Fig Protease inhibitors prevent breakdown of histatins and cytokines during incubation with chronic wound extract. (XLSX)

S4 Fig Dose response of Hst1 and PIC in the presence of CWE. (XLSX)

S5 Fig An 8 h pulse of peptides stimulates fibroblast migration to the same extent as continuous 96 h exposure. (XLSX)

## Abbreviations

CWE: chronic wound extract
Hst: histatin
cHst1: cyclic Hst1
mad-Hst1: minimal active domain of Hst1
PIC: protease inhibitor cocktail
rh: recombinant human
MMPs: matrix metalloproteinases
